# Deep Learning Signal Discrimination for Improved Sensitivity at High Specificity for CMOS Intraoperative Probes

**DOI:** 10.1109/TRPMS.2021.3098448

**Published:** 2021-07-19

**Authors:** Joshua Moo, Paul Marsden, Kunal Vyas, Andrew J. Reader

**Affiliations:** School of Biomedical Engineering and Imaging SciencesKing’s College London London SE1 7EH U.K.; Research DepartmentLightpoint Medical Ltd. Chesham HP5 1PE U.K.

**Keywords:** Cancer surgeries, CMOS, convolutional neural network (CNN), internal conversion (IC) electrons, intraoperative beta probes

## Abstract

The challenge in delineating the boundary between cancerous and healthy tissue during cancer resection surgeries can be addressed with the use of intraoperative probes to detect cancer cells labeled with radiotracers to facilitate excision. In this study, deep learning algorithms for background gamma ray signal rejection were explored for an intraoperative probe utilizing CMOS monolithic active pixel sensors optimized toward the detection of internal conversion electrons from $^{99m}$Tc. Two methods utilizing convolutional neural networks (CNNs) were explored for beta-gamma discrimination: 1) classification of event clusters isolated from the sensor array outputs (SAOs) from the probe and 2) semantic segmentation of event clusters within an acquisition frame of an SAO which provides spatial information on the classification. The feasibility of the methods in this study was explored with several radionuclides including ^14^C, ^57^Co, and $^{99m}$Tc. Overall, the classification deep network is able to achieve an improved area under the curve (AUC) of the receiver operating characteristic (ROC), giving 0.93 for ^14^C beta and $^{99m}$Tc gamma clusters, compared to 0.88 for a more conventional feature-based discriminator. Further optimization of the lower left region of the ROC by using a customized AUC loss function during training led to an improvement of 31% in sensitivity at low false positive rates compared to the conventional method. The segmentation deep network is able to achieve a mean dice score of 0.93. Through the direct comparison of all methods, the classification method was found to have a better performance in terms of the AUC.

## I. Introduction

One of the main challenges faced in cancer resection surgeries is the difficulty in delineating the boundary between cancerous and healthy tissue, leading to suboptimal surgical outcomes due to the overestimation of the extent of tumor margins in order to achieve complete tumor removal, which inadvertently causes the excessive removal of healthy tissue. On the other hand, in cases where tumor excision is incomplete, subsequent resection operations and extensive postoperative adjuvant radiotherapy are required which in turn will reduce patient survival rates [1]. Current margin evaluation methods involve the pathological analysis of biopsies taken during surgery, where the results can take up to weeks to be able to inform if re-excision is required [2]. Therefore, there is a need for the real-time detection of cancerous tissue during surgery which can be achieved through intraoperative technology.

Optical image-guided cancer surgery which involves the detection of photons emitted by tumor-specific fluorescence agents was also presented as a solution for real-time tumor margin delineation. However, the need for the development of these tumor-specific agents has limited clinical adoption due to: 1) the efficiency of the agents in being able to reach and remain at the target location; 2) degradation in signal localization due to absorption and scattering of light in tissue; and 3) lack of financial incentives for the production of these agents for diagnostic purposes [3].

The use of gamma and beta emitting radiopharmaceuticals which accumulates within cancer cells in conjunction with intraoperative probes presents a potential solution for tumor localization during surgery. Initially, several gamma sensitive intraoperative probes were designed to target $\gamma $ emitting radiotracers due to the large range of gamma rays in tissue, which allows the detection of tumors that are located deep under the tissue surface. However, these attempts were found to be suboptimal due to the susceptibility of the gamma probes to the highly penetrating gamma rays from distant organs due to the nonspecific uptake of the radiotracer, degrading the tumor-to-background signal ratio which leads to the ambiguity of tumor location [4], [5].

Research interests have then shifted toward beta sensitive intraoperative probes due to the short range of electrons and positrons in tissue, which presents as an advantage over gamma probes since signal is detected only when placed in close proximity to the source. Moreover, the effects associated with the nonspecific radiotracer uptake are diminished due to lower signal interference from distant sources, which improves the spatial selectivity of the probe [6], [7]. Several beta intraoperative probes have been proposed with designs that are optimized toward the rejection of background gamma rays by subtraction or coincidence detection through the implementation of multiple detectors within a single probe which imposes physical limitations on the design of the probe [6], [8], [9]. For example, Yamamoto *et al.* [9] proposed a beta intraoperative probe involving a phoswich detector composed of a plastic scintillator and a high Z scintillator, bismuth germanate (BGO). The probe employs pulse shape discrimination for positron detection and rejection of background gamma events through coincident detection of a positron and one of its corresponding 511-keV annihilation photons in conjunction with the different decay times of the scintillators. Therefore, the discrimination method is only restricted to ^18^F-fluorodeoxyglucose (FDG) guided surgeries. On top of that, shielding of the probe is required to reduce the susceptibility to background gamma rays which would inadvertently compromise the sensitivity of the probe for positrons.

Using CMOS sensors for the detection of ionizing radiation have been investigated in [10], and more specifically beta emissions in [11]. These studies have shown the feasibility of using CMOS sensors for the detection of $\mathrm {\beta } ^{-}$ emissions from ^90^Sr and ^90^Y (pure $\mathrm {\beta } ^{-}$ emitting sources) phantoms under realistic clinical conditions through the implementation of a clustering algorithm for event detection, which considers a neighborhood of pixels around high intensity seed pixels. Although the sensitive (epitaxial) layer of CMOS sensors is generally sufficiently thin (orders of a few micrometers) to ensure low gamma ray interaction probabilities, there is still a finite detection efficiency which means there will be background gamma ray signal contamination. Therefore, there is still a need for the active rejection of background gamma ray of lower energies through the analysis of the event clusters detected by the CMOS sensor. The use of convolutional neural networks (CNNs): 1) utilizes their ability to learn and extract appropriate features from training examples for the specific classification of beta and gamma clusters and 2) obviates the need for hand-crafted features which relies on the incorrect assumption that such features are able to capture all relevant information for the classification task [12].

**Fig. 1.**
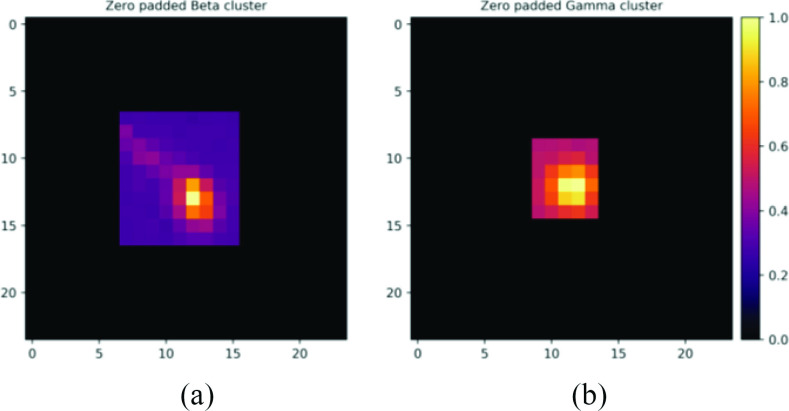
(a) Example of beta cluster from ^14^ C and (b) gamma cluster from $^{99m}$Tc. The event clusters are cropped based on the size of their bounding box and zero padded.

In this study, two deep learning architectures for background gamma ray rejection are proposed for an intraoperative probe utilizing CMOS monolithic active pixel sensors optimized toward the detection of internal conversion (IC) electrons from $^{99m}$Tc, which is more readily available and cost effective. On top of that, $^{99m}$Tc also has a longer half-life of 6 h which allows for more flexible scheduling of surgeries. The charge distribution over several detector pixels caused by the emissions can be isolated (Fig. 1) and the rejection of gamma signal is possible with only a single detector if discrimination between gamma and beta clusters can be achieved. Therefore, methods utilizing CNNs were explored for beta–gamma discrimination: 1) classification of event clusters isolated from the sensor array outputs (SAOs) and 2) semantic segmentation of event clusters within each SAO acquisition frame.

## II. Methods and Materials

### A. Detector

**Fig. 2.**
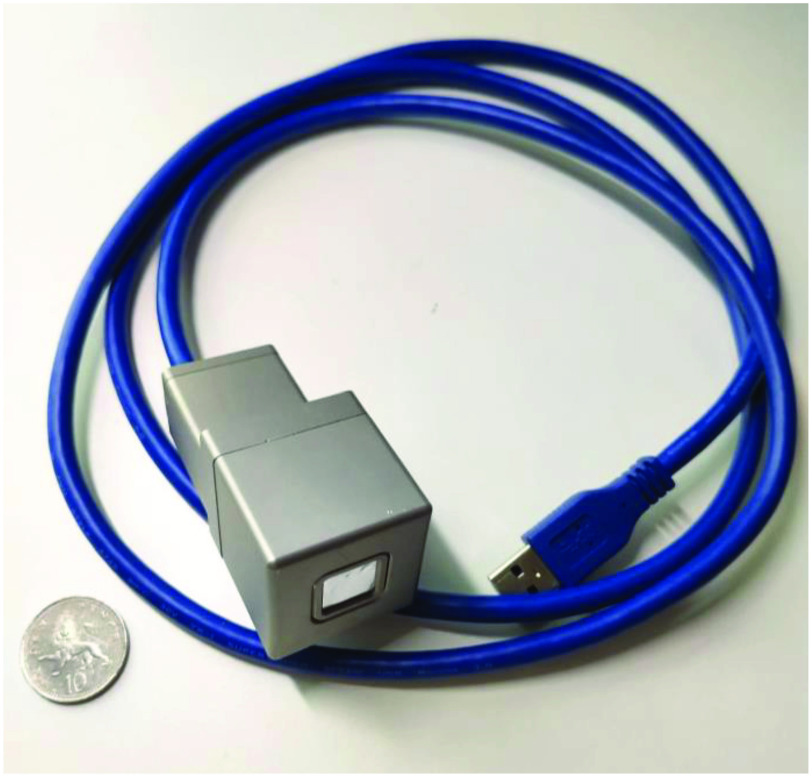
Prototype CMOS intraoperative probe developed by lightpoint medical.

A prototype intraoperative probe (Fig. 2) developed by Lightpoint Medical utilizes a CMOS sensor ( $480\,\,\mathrm {\times }$ 640 pixels, pixel size $6\,\,\mu \text{m}\,\,\mathrm {\times }\,\,6\,\,\mu \text{m}$, and a sensitive area of 3.84 mm $\mathrm {\times }\,\,2.99$ mm). The thickness of the sensitive layer, which is the epitaxial layer of the CMOS sensor is approximately $4~\mu \text{m}$ (a similar model was used in [11]) and is capable of achieving 60 frames/s. The detection efficiency of a CMOS sensor with a similar sensitive layer thickness is reported to be 2% for gamma rays with energies higher than 20 keV [11]. The probe is connected to a PC via USB for image acquisition, transmission, processing, and analysis. To account for the dark current or electronic noise of each pixel, several blank frames are initially acquired to obtain an average dark image which is then subtracted from each image acquired in the presence of a radioactive source.

### B. Sources and Cluster Detection

A labeled dataset, where the identity of the clusters is known, is required for training the CNNs for beta–gamma discrimination. Therefore, SAOs from pure $\beta ^{-}$ (^14^C) and $\gamma $ (^57^Co) emitting radionuclides were obtained which allows the clusters to be identified. SAOs containing only $^{99m}$Tc gammas were acquired by using a 2-mm PMMA film to shield against IC electrons. The emission types and probabilities of the radionuclides used in this study are shown in Table I. $\beta ^{-}$ clusters from ^14^C was used as a surrogate for $^{99m}$Tc IC electrons due to their similar emission energies.

**TABLE I:** List of Emissions for Radionuclides Used

Radioisotope	Emission type	Emission probability (%)	Energy (keV)
^14^C	$\beta^{-}$	100	156 (max)
^57^Co	$\gamma $	91	136
	$\gamma$	9	122
^99m^Tc	$\gamma$	88	140
	IC electron	12	119–140^*^

^*^ Energies are discrete and dependent upon the binding energy of the electron shell

A threshold is applied to the dark current-corrected image which results in a binary image containing the clusters. Connected components labeling (8-connectivity) is then applied to the binary image which allows the position and bounding box size of each event cluster within a frame to be determined. Using this information, the clusters are isolated from the original dark current-corrected image which is then saved to form a dictionary. SAOs with ^14^C were used to generate a beta cluster dictionary, whereas SAOs from ^57^Co and $^{99m}$Tc with PMMA were used to generate the respective gamma cluster dictionaries. Overall, 123 107 ^14^C beta clusters, 20 359 ^57^Co gamma clusters, and 9887 $^{99m}$Tc gamma clusters were obtained.

### C. Classification (Models C1 and C2)

**Fig. 3.**
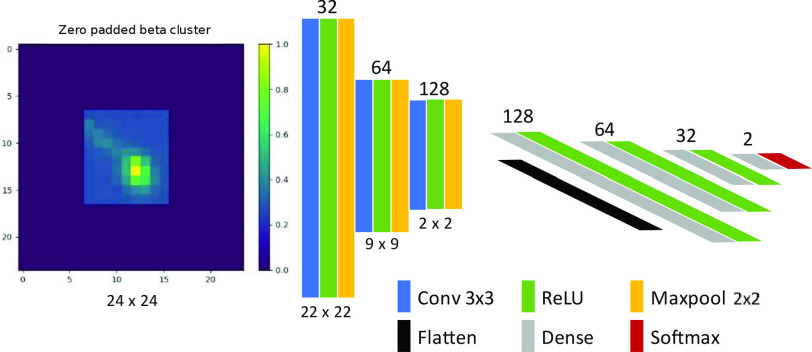
Model architecture for classification of beta and gamma clusters.

The individual clusters in the dictionaries were initially zero padded to an image patch of $24\,\,\mathrm {\times }\,\,24$ pixels in order to account for the variable cluster size which allows for a consistent input to the network. The clusters patches were also normalized to values between 0 and 1. The overall model architecture (Fig. 3) consists of three convolutional layers with kernels of size $3\,\,\mathrm {\times }\,\,3$, followed by a rectified linear unit (ReLU, a commonly used nonlinear activation function which sets negative values to zero while retaining all positive values [13]) activation at each layer. Each convolutional layer is also followed by a max pooling layer with $2\,\,\mathrm {\times }\,\,2$ kernel size for feature dimension reduction. The filter maps after these feature extraction operations are then flattened to provide a 1-D feature vector to 4 fully connected layers with 128, 64, 32, and 2 nodes, respectively. A softmax activation (which normalizes a vector into a probability distribution [14]) is applied at the output layer to provide a probabilistic score for each class and the loss function used is the categorical cross-entropy (CE)

\begin{equation*} \textrm {CE}=-\frac {1}{N}\sum ^{N}_{n=1}{\sum ^{C}_{c=1}{y_{n,c}{\mathrm {log} p_{n,c}~}}}\tag{1}\end{equation*}

which is calculated between the output prediction, $p$, and one-hot encoded ground truth, $y$, across the number of samples, $N$, and the number of classes, $C$. The classification architecture is adapted from LeNet-5 [12] which was originally designed for handwriting recognition. Given that there are only two classes for this application, the number of layers and channels was reduced from the original LeNet-5 architecture to the facilitate training time. 60% of the total clusters were used for training, 20% for validation, and 20% for testing. Model C1 is trained and tested with ^14^C beta and ^57^Co gamma clusters and model C2 is trained and tested with ^14^C beta and $^{99m}$Tc gamma clusters. The reason for training the two different models is to evaluate the ability of the network to discriminate between beta and gamma clusters from pure sources as well as when $^{99m}$Tc is used, which is the radiotracer of interest in this application. Due to the difficulty in obtaining SAOs containing only IC electrons from $^{99m}$Tc, the beta clusters from ^14^C were used in model C2 since the energies of these betas are comparable to the IC electrons. A receiver operating characteristic (ROC) curve was generated for both models where the true positive rate (TPR) is plotted against the false positive rate (FPR). The positive class in the ROC curves corresponds to the beta class.

**Fig. 4.**
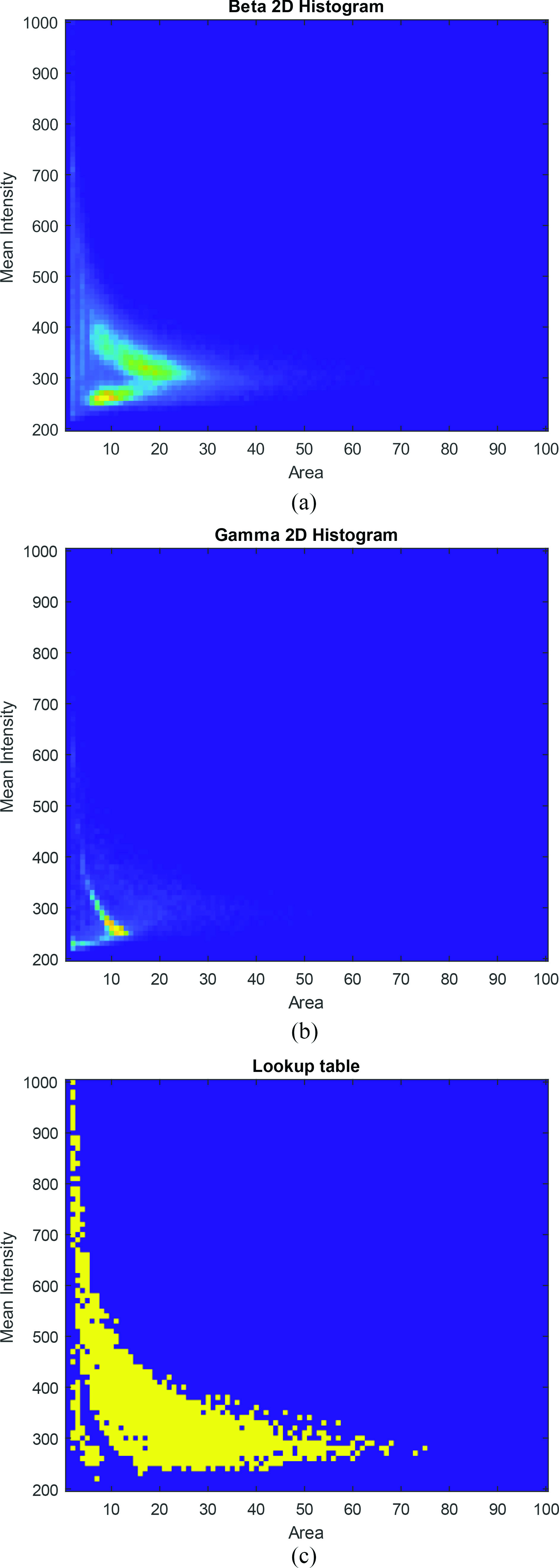
2-D histograms of mean intensity and area of (a) beta and (b) gamma events. (c) Lookup table generated from both histograms.

The area under the ROC curves (AUCs) is used as a measurement for the performance of the classifiers, where an AUC of 1 corresponds to a perfect classifier. The AUC of the models were also compared to a feature-based lookup table classifier developed by Lightpoint Medical which is implemented in MATLAB (The Mathworks, Inc.). Initially, 2-D histograms for beta [Fig. 4(a)] and gamma [Fig. 4(b)] clusters based on their mean intensity and area (the product of which is proportional to the total energy deposited) were generated, which are then normalized by the total number of events to give a probability distribution. The beta-events histogram is divided by the gamma-events histogram to obtain a selectivity map, where the lookup table [Fig. 4(c)] can be generated such that clusters that fit into regions on the map that shows selectivity above a threshold will be classified as betas. ^14^C beta and $^{99m}$Tc gamma clusters were also tested on model C1 to evaluate the possibility of applying transfer learning during the training process such that a model trained on pure beta and gamma emitting sources is able to distinguish between gamma and IC electron signals from $^{99m}$Tc.

In this application, misclassifying gamma clusters as beta clusters would falsely indicate the presence of cancerous tissue within the region of interest. Therefore, the cost of false positives is higher than false negatives which means a decision threshold will be selected such that the classifiers will be operating at a low FPR or high specificity. Given that the CE loss function does not take into account these misclassification costs during training, there is a need for further optimization of the classifier operating at high specificity, which corresponds to the lower left region of the ROC curve. Therefore, an alternative loss function which utilizes the equivalency of the Wilcoxon-Mann-Whitney (WMW) statistic and the AUC was used to train the model to directly maximize the AUC of the lower left region of the ROC curve [15]. The WMW statistic is approximated by the differentiable function

\begin{align*} R\left ({x_{i},y_{j}}\right)=\begin{cases} {\left ({-\left ({x_{i}-y_{j}-\gamma }\right)}\right)}^{p}, \quad x_{i}-y_{j}< \gamma \\ 0, \qquad \qquad \qquad \qquad \textrm {otherwise} \end{cases}\tag{2}\end{align*}

where $x_{i}$ and $y_{j}$ are the probabilistic scores for the positive and negative class, respectively. The hyperparameters $\gamma $ and $p$ are set to 0.7 and 2, respectively, which corresponds to the optimal values determined through grid search in [15]. The loss function $L _{1}$ can then be formulated as

\begin{equation*} L_{1}=\sum ^{m}_{i=1}{\sum ^{n}_{j=1}{R\left ({x_{i},y_{j}}\right)}}\tag{3}\end{equation*}

where $m$ and $n$ are the number of positive and negative samples, respectively. Positive samples with scores that are higher than negative samples by a margin $\gamma $ have no contribution to the loss function. The loss function can then be extended to focus on the lower left region of the ROC curve by mapping the probabilistic scores $s$ obtained from the final softmax layer of the classification network function

\begin{align*} f\left ({s}\right)=\begin{cases} {\left ({s-{\mathrm {\mu }}_{s}}\right)}^{\alpha },& s > {\mathrm {\mu }}_{s} \\ 0,& \textrm {otherwise} \end{cases}\tag{4}\end{align*}

where ${\mathrm {\mu }}_{s}$ is the mean of the probabilistic scores and the hyperparameter $\alpha $ is set to 1.1, which is again found to be optimal through grid search. Thus, the network is then trained using the loss function $L _{2}$

\begin{equation*} L_{2}=\sum ^{m}_{i=1}{\sum ^{n}_{j=1}{R\left ({f(x_{i}),f(y_{j})}\right)}}\tag{5}\end{equation*}

where training is only focused on samples with scores higher than the mean score. This concept of explicitly selecting data that is within the region of interest on the ROC curve for training is also demonstrated in [16]. In this case, clusters assigned with a high probabilistic score by the network are either easily identifiable beta clusters or difficult gamma clusters. Learning to distinguish between these clusters would contribute the most to improving the AUC at the lower left region of the ROC.

### D. Segmentation (Models S1 and S2)

Semantic segmentation was also explored as a method to directly label the beta and gamma clusters within an acquisition frame of an SAO in one step by assigning a class label to each pixel. The lack of ground-truth SAOs containing both beta and gamma clusters motivated the synthesis of SAOs using clusters from measured dictionaries. Initially, the clusters are added to a zero image and the remaining zero values in the resulting image are then replaced with values sampled from a fitted normal distribution of background intensities where the mean and variance are determined from real images acquired by the detector. The synthesized SAOs are of size $240\,\,\mathrm {\times }\,\,320$ (smaller than the native SAO to reduce computational costs). Finally, the images are then normalized to values between 0 and 1 which is then used as the input to the segmentation network.

**Fig. 5.**
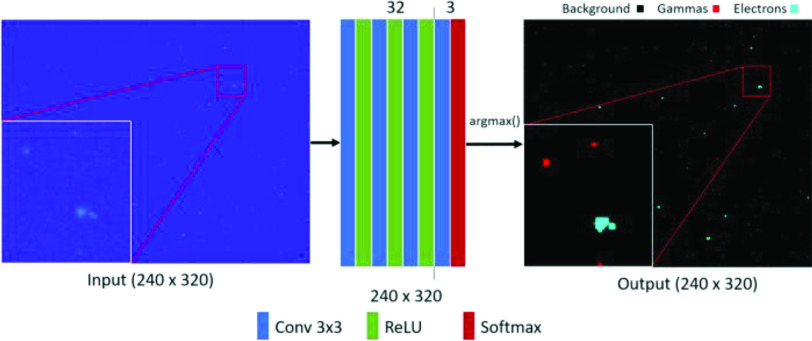
Model architecture for semantic segmentation. A frame of the SAO is fed into the network where the event clusters will be labeled and segmented.

The network architecture for segmentation is shown in Fig. 5, consisting of three convolutional layers with 32 kernels of size $3\,\,\mathrm {\times }\,\,3$ with ReLU activation at each layer. This is then followed by a convolutional layer consisting of three kernels of size $3\,\,\mathrm {\times }\,\,3$ with softmax activation. The output consists of an image with three channels where each pixel contains three probabilistic scores for the background, beta, and gamma class. The class with the highest score is then assigned to the pixel. The targets are $240\,\,\mathrm {\times }\,\,320\,\,\mathrm {\times }\,\,3$ images where each channel corresponds to a binary image for each class and the loss function used is the generalized dice loss (GDL) which is reported to have better performance over the more conventionally used CE loss in the presence of class imbalance [17], which in this case there are more pixels in the SAO belonging to the background class compared to either the beta/gamma class

\begin{equation*} \textrm {GDL}=1-2\frac {\sum ^{C}_{c=1}{w_{c}}\sum ^{N}_{n=1}{y_{c,n}\mathrm {\cdot }p_{c,n}}}{\sum ^{C}_{c=1}{w_{c}}\sum ^{N}_{n=1}{y_{c,n}+p_{c,n}}}\tag{6}\end{equation*}

with

\begin{equation*} w_{c}=\frac {1}{{\left ({\sum ^{N}_{n=1}{y_{c,n}}}\right)}^{2}}\tag{7}\end{equation*}

where $y$ corresponds to the ground truth or gold standard segmentation labels and $p$ corresponding to the predicted probabilistic values, calculated across $N$ number of pixels and $C$ number of classes. The contribution to the loss function from each class is weighted by the inverse of its area $w_{c}$ to provide invariance to class imbalance. Model S1 is trained and tested with SAOs containing ^14^C beta and ^57^ Co gamma clusters and Model S2 is trained and tested with SAOs containing ^14^ C beta and $^{99m}$Tc gamma clusters. A total of 500 images were synthesized with 60% used for training, 20% for validation, and 20% for testing. To be able to provide a performance comparison in terms of beta–gamma discrimination with the classification methods mentioned above, an ROC curve was generated based on the scores of the clusters. To achieve this, the beta and gamma scores for each cluster are extracted and a softmax activation is applied. The mean of the resulting beta scores of each cluster are then used to generate the ROC curve. To evaluate whether the number of patches used in this study is sufficient to optimize the discrimination task, model C2 was retrained at different fractions of the total number of patches obtained experimentally and the AUC metric on the test set was recorded.

## III. Results

### A. Classification

**Fig. 6.**
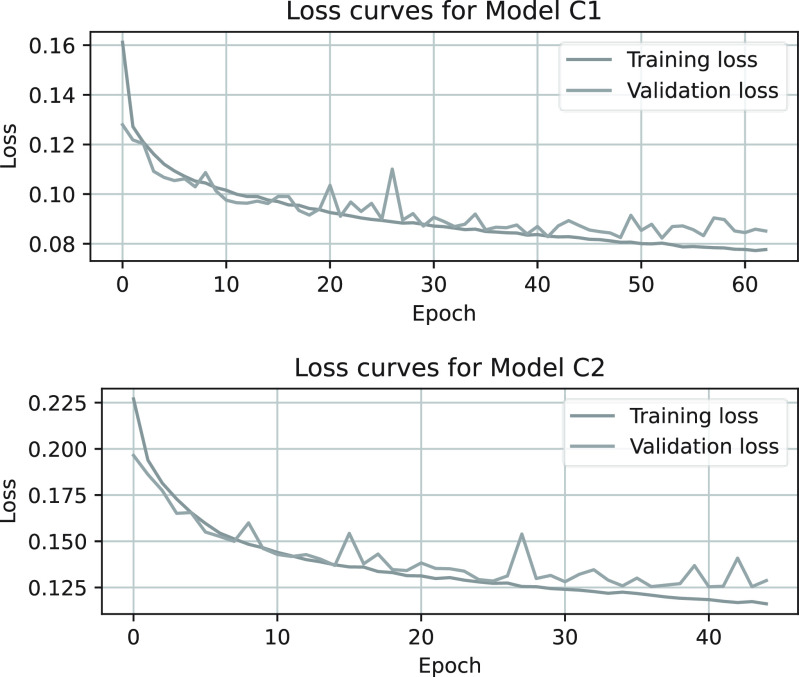
Training and validation loss as a function of the number of epochs. Early stopping was determined through monitoring the validation loss with a patience of 10 epochs.

**Fig. 7.**
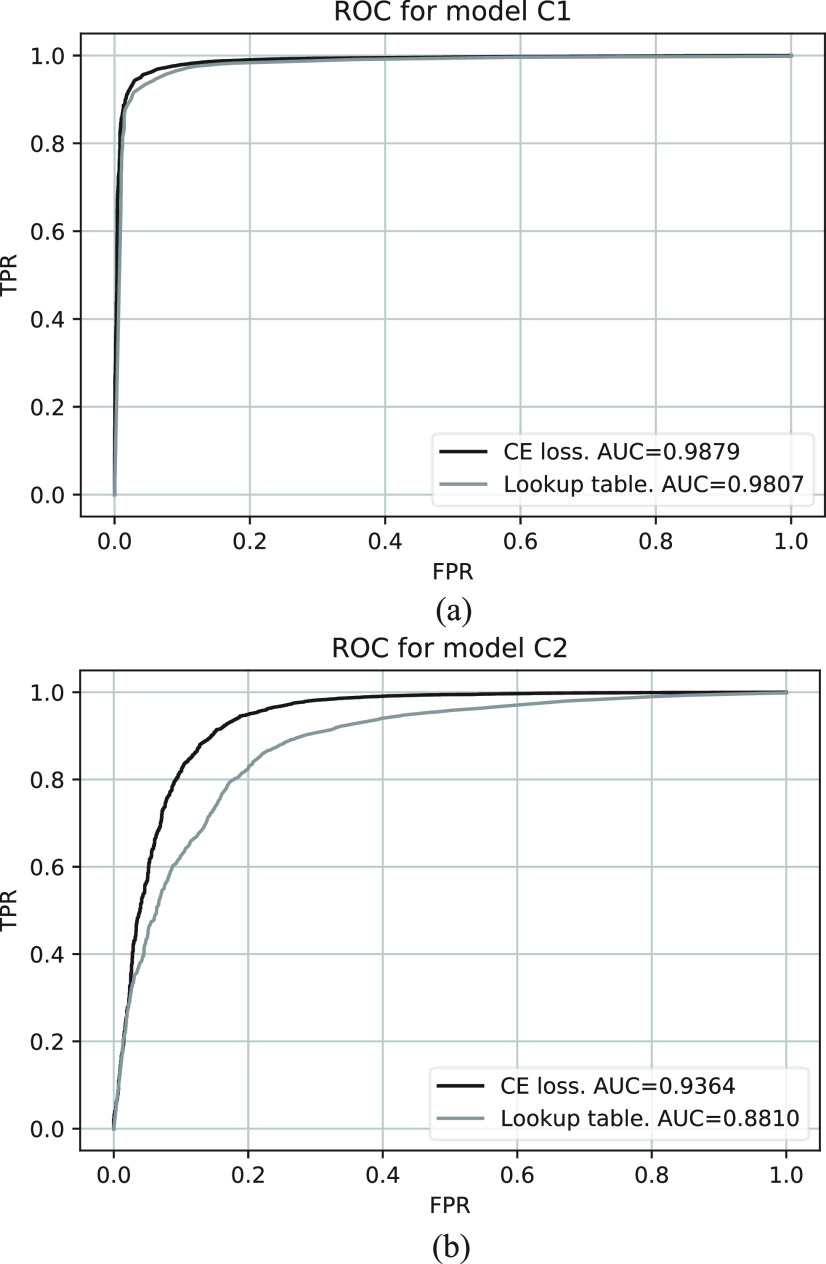
Comparison of ROC curves between CNN classifier and lightpoint lookup table classifier. (a) ROC for Model C1, trained and tested with ^14^C beta and ^57^Co gamma clusters. (b) ROC for Model C2, trained and tested with ^14^C beta and $^{99m}$Tc gamma clusters.

**Fig. 8.**
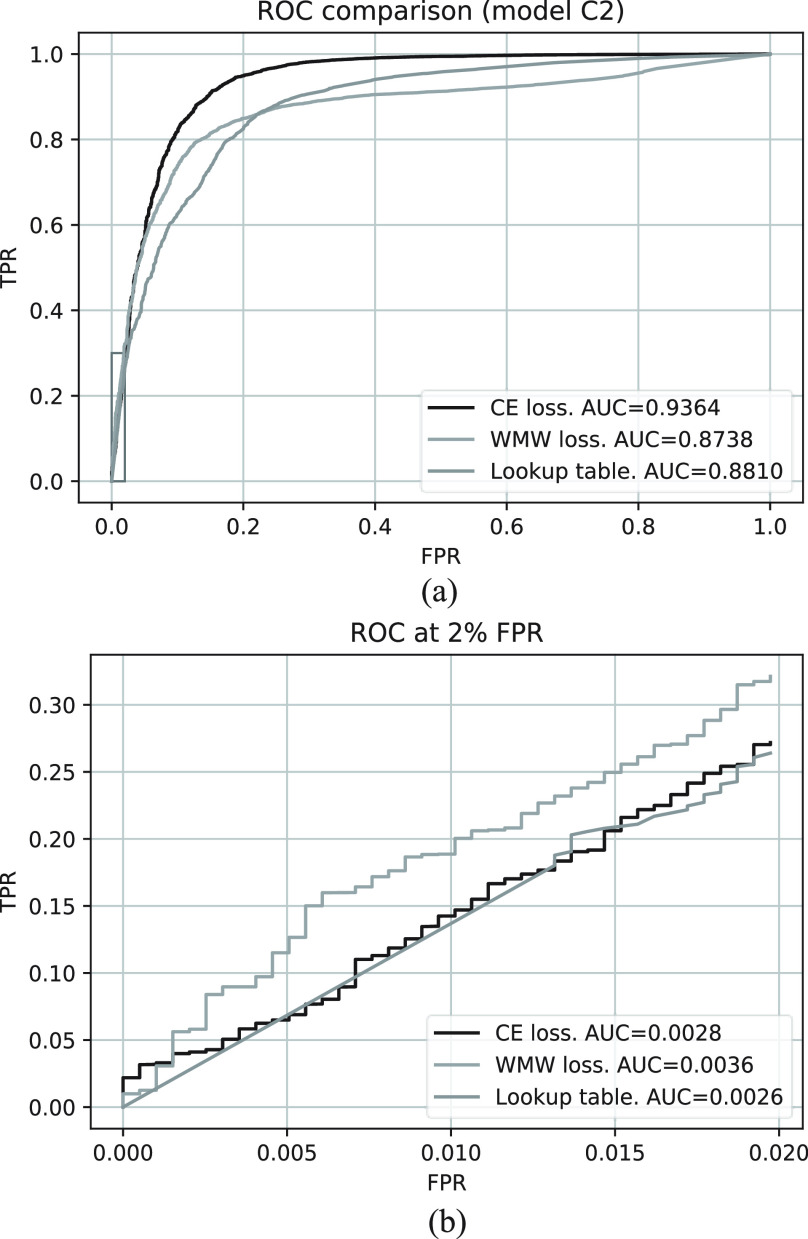
(a) Comparison of overall ROC between categorical CE loss and WMW loss with model C2. (b) Comparison of lower left part of the ROC. AUC values reported are at 2% FPR.

**Fig. 9.**
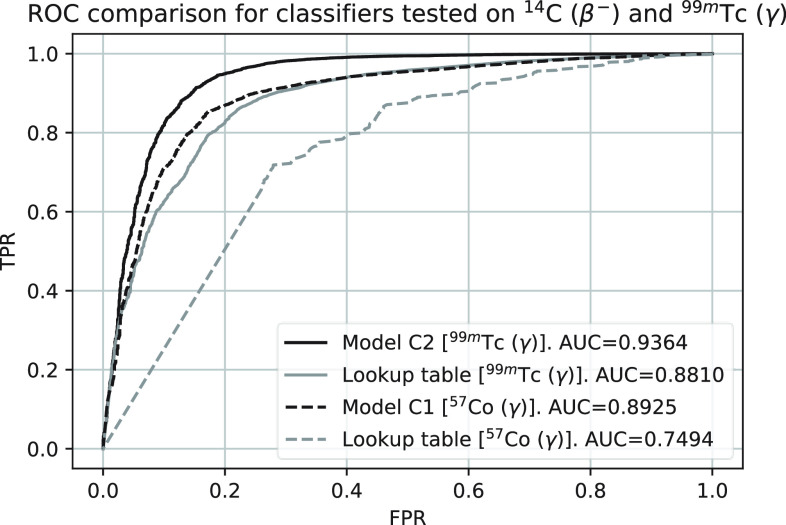
Comparison of ROC for all classifiers tested on ^14^C beta and $^{99m}$Tc gamma clusters. All classifiers were trained on ^14^C beta clusters and the gamma clusters depicted in the square brackets for each classifier.

**Fig. 10.**
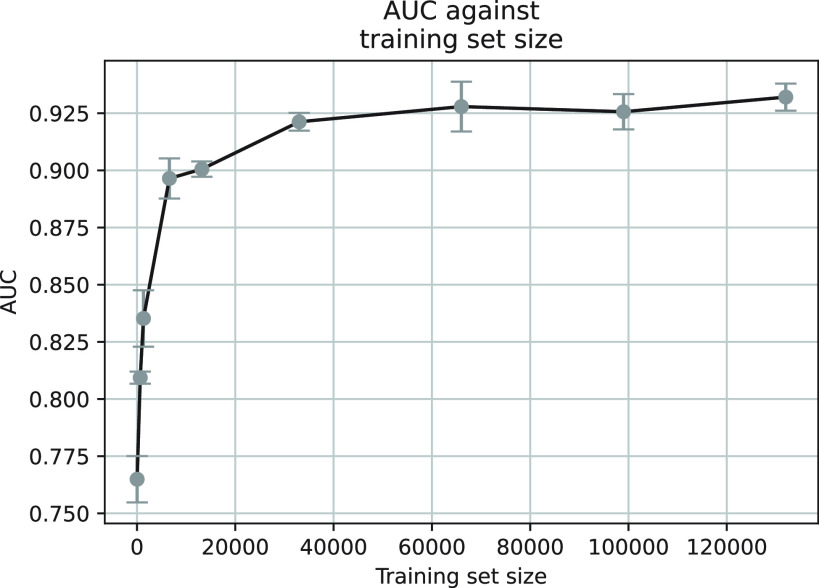
Training set size grid search conducted with model C2 trained using CE loss. The mean and standard deviation values are calculated across five repeats.

The training and validation losses for models C1 and C2 are shown in Fig. 6. The ROC curves for both models are shown in Fig. 7 where the AUCs for model C1 and C2 are 0.988 and 0.936, respectively. Fig. 8 shows a comparison between the CE and WMW loss functions with model C2. Overall, model C2 trained with the WMW loss achieved the lowest AUC [Fig. 8(a)]. However, if we consider the ROC up to 2% FPR (lower-left region of the ROC), WMW loss achieved the best performance in terms of the AUC when compared to either using CE or the lookup table [Fig. 8(b)]. Fig. 9 shows the ROC curves for all classifiers tested with ^14^C beta and $^{99m}$Tc gamma clusters. A lower AUC is observed with model C1 compared to model C2, however, it is still comparable to the lookup table trained with ^14^C beta and $^{99m}$Tc gamma clusters. The AUC score as a function of training set size with model C2 is reported in Fig. 10.

### B. Segmentation

**Fig. 11.**
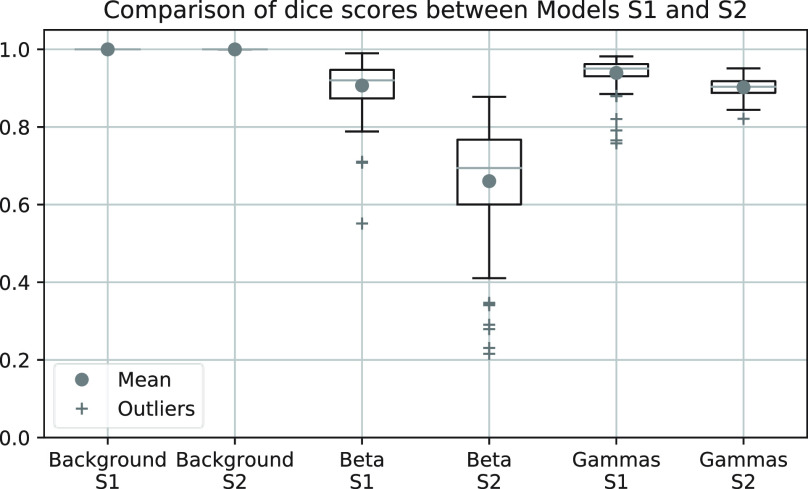
Comparison of dice scores for each class between models S1 and S2.

**Fig. 12.**
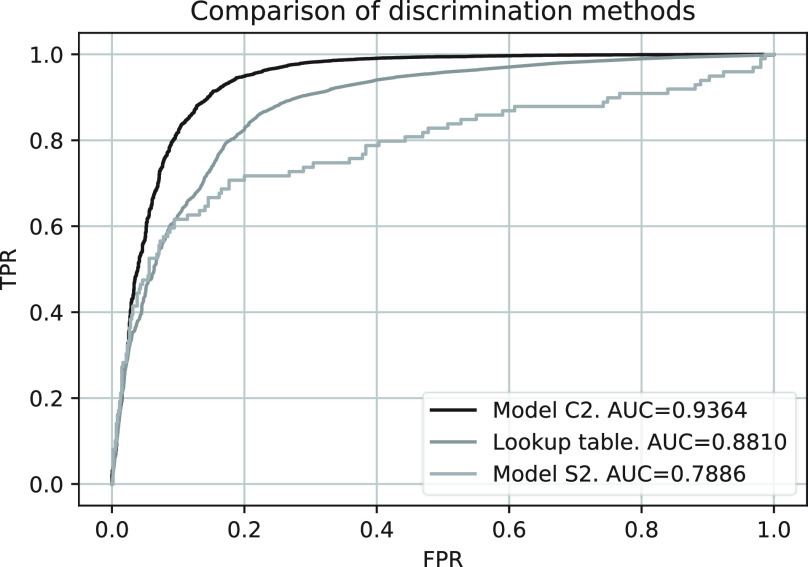
Comparison of ROC between models C2 and S2.

The dice scores of each individual class were monitored as the performance metric and are shown in Fig. 11. The dice scores for the background class are close to unity for both models. However, lower dice scores for the gamma and beta class are found with model S2 when compared to model S1. The ROC curve generated based on the mean beta scores of the clusters from segmentation with model S2 is compared with model C2 in Fig. 12. A lower AUC is observed with model S2.

## IV. Discussion and Conclusion

Both deep learning methods have shown promising results for beta–gamma discrimination. Better performance in terms of the AUC is observed with both models C1 and C2 compared to the lookup table classifier, as the classification network learns many features for itself, compared to the two handcrafted features which are the mean intensity and area of the clusters. The lower performance observed with Model C2 compared to C1 could be due to the presence of IC electrons in the $^{99m}$Tc gamma dictionary and a higher degree of class imbalance between ^14^C beta and $^{99m}$Tc gamma clusters. The performance with model C1 when tested on $^{99m}$Tc gamma clusters shows the possibility of implementing transfer learning during the training process such that the classification network can be applied to discriminating the emissions from $^{99m}$Tc. This would be especially beneficial due to the difficulty in isolating the IC electron signals from $^{99m}$Tc for training the classifier.

The need to optimize the lower left region of the ROC curve is essential since misclassifying gamma signals as beta signals would lead to unhelpful ambiguity of the tumor location. The use of the WMW loss allowed the direct optimization of the AUC as well as improvement in sensitivity at high specificity. The model trained with WMW loss achieved approximately an improvement of 31% in AUC compared to the model trained with CE loss up to an FPR of 0.02, which would be within the typical range of operation in a surgical environment. At approximately 0.03 FPR, the model trained with CE loss begins to outperform the WMW loss in terms of sensitivity which is expected due to the mapping applied to the samples in (4). Therefore, this presents a tradeoff between optimization at high and low specificity.

Increasing the training set size shows an improvement in performance in terms of the AUC is seen in Fig. 10. With a training set size above 60 000, the AUC begins to plateau which shows a point of diminishing returns has been reached and further increase in training set size will yield a smaller increase in performance with a longer training time. This also shows that the total number of patches used in this study is sufficient for the discrimination task.

**Fig. 13.**
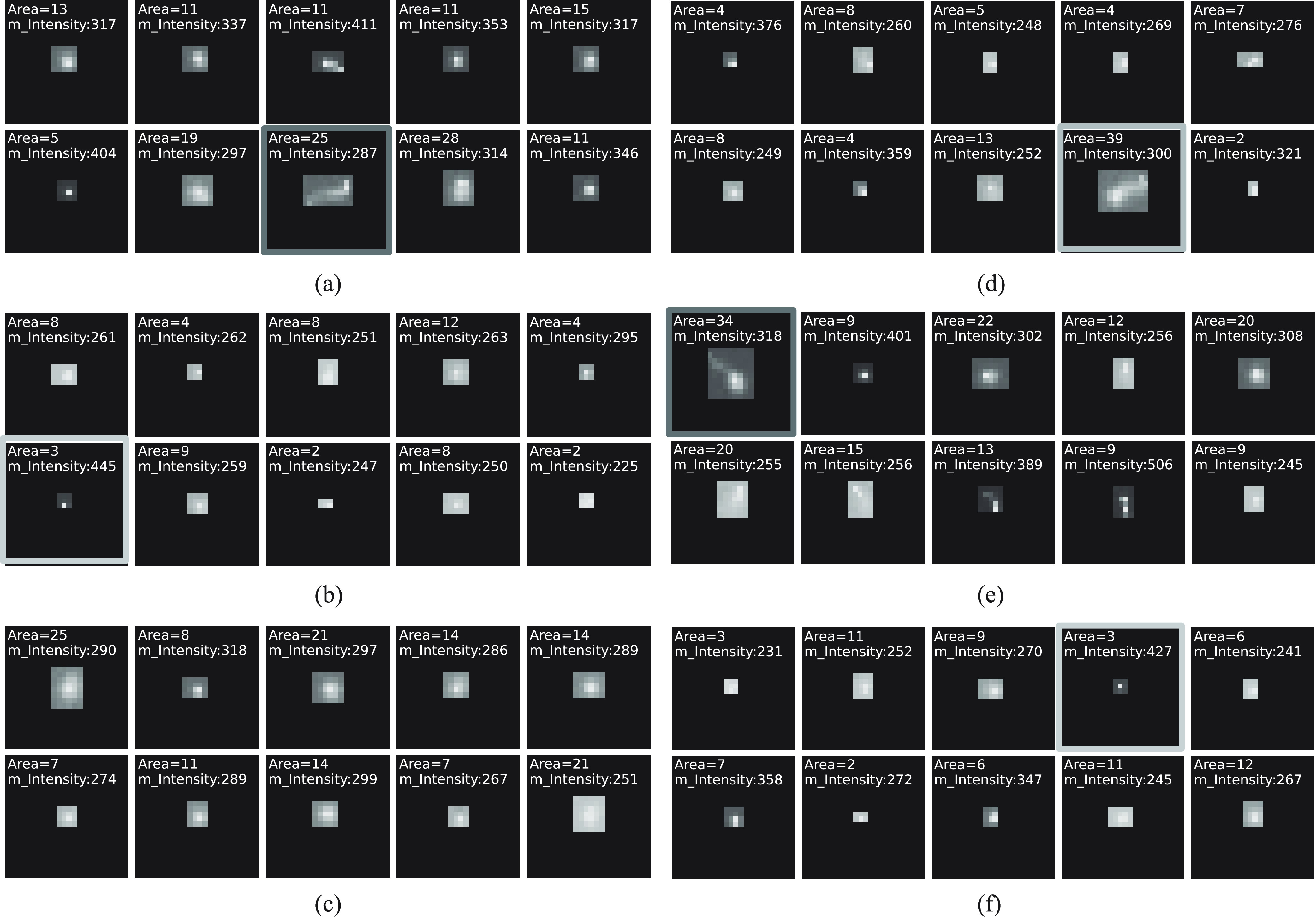
(a) and (b) Examples of misclassifications made by both CNN and lookup table classifiers. (c) and (d) Examples of misclassifications made by the lookup table but not the CNN. (e) and (f) Examples of beta and gamma clusters, respectively. The gamma cluster in row 2, column 3 in (a) has similar appearance and physical properties to the beta cluster in row 1, column 1 of (e) (highlighted in red). A similar comparison can be made between the beta cluster in row 2, column 1 of (b) and the gamma cluster in row 1, column 4 of (f) (highlighted in yellow). An apparent example of a beta cluster in (d) was misclassified by the lookup table but not the CNN classifier (highlighted in green).

Examples of misclassifications and each type of clusters are shown in Fig. 13. These cases are primarily due to the clusters having similar appearances and physical properties to clusters in the other class. This could be due to the presence of noise in the training data labels, which causes the ambiguity of the identity of the cluster. An apparent example of a beta cluster in Fig. 13(d) (highlighted in green) was misclassified by the lookup table but not the CNN classifier. This demonstrates that the features learned by the CNN for the classification were able to outperform the hand-crafted features used to generate the lookup table.

The dice scores with semantic segmentation for the background class demonstrates the ability of the networks to segment the clusters from the SAOs. The dice scores for the beta and gamma class have also shown that discrimination between the two can be achieved in one step. However, better performance in terms of the AUC is observed for the classification method because more information is available, requiring only one classification of many pixels, compared to requiring classification for each and every pixel in the SAO for the segmentation network. Overall, through the direct comparison of both methods, the classification network yielded better performance in beta–gamma discrimination.

In conclusion, the deep learning-based gamma signal rejection methods in this study have shown improved performances compared to a deterministic machine-learned method as well as further improvement at low FPRs which is essential for clinical situations.
